# Characteristics associated with first anti-seizure medication prescribed in a cohort of adults with newly diagnosed epilepsy

**DOI:** 10.1016/j.seizure.2026.02.007

**Published:** 2026-02-05

**Authors:** Leah J. Blank, Rachelle Morgenstern, Kenneth Boockvar, Nihal Mohamed, Nathalie Jetté

**Affiliations:** a Department of Neurology, Division of Health Outcomes & Knowledge Translation Research, Icahn School of Medicine at Mount Sinai, New York, NY, USA; b Department of Population Health and Policy, Institute for Healthcare Delivery, Icahn School of Medicine at Mount Sinai, New York, NY, USA; c Department of Medicine, Division of Gerontology, Geriatrics and Palliative Care, University of Alabama at Birmingham, Birmingham, AL, USA; d Geriatrics Research, Education, and Clinical Center, Birmingham VA Health Care System, AL, USA; e Department of Urology, Icahn School of Medicine at Mount Sinai, New York, NY, USA; f Department of Clinical Neurosciences, University of Calgary, Calgary, AB, Canada

**Keywords:** Antiepileptic, AED, Prescribing, Polypharmacy, Predictor

## Abstract

**Background:**

Anti-seizure medication (ASM) is the primary treatment modality in epilepsy. There exist evidence-based recommendations published by the American Academy of Neurology and American Epilepsy Society for ASM selection in epilepsy, but these medication recommendations are inconsistently followed. We sought to examine predictors of recommended first ASM in newly diagnosed adults with epilepsy.

**Methods:**

We conducted a retrospective cohort study of adults (≥18 years) newly diagnosed with epilepsy in New York, identified using validated ICD-CM codes, for the period 2011 to 2019. The primary outcome of interest was use of an AES/AAN guideline-informed ASM, and exposures of interest included patient characteristics (e.g. age), provider characteristics (e.g. specialty) and structural characteristics (e.g., practice setting). Multivariable Poisson regression for risk ratios modeled the probability of being prescribed a neutral/recommended or non-recommended ASM, adjusting for covariates.

**Results:**

2340 adults with newly diagnosed epilepsy were prescribed an ASM within 1-year. The most frequently prescribed ASM was levetiracetam (45.5%), which aligns with recommendations. However, 39% were prescribed a non-recommended medication. The prescription of a recommended ASM was associated with older age at diagnosis (relative risk (RR) 1.01; 95% confidence interval (CI) 1.00–1.01), Black race (RR 1.12; 95% CI 1.03–1.23), being in a relationship (vs. divorced/separated/single or widowed) (RR 1.13; 95% CI 1.06–1.22) and a history of stroke (RR 1.19; 95% CI 1.05–1.34). Prescriptions from physician trainees (vs. non-trainees) were more likely to align with recommended ASMs (RR 1.15; 95% CI 1.06–1.25), with no differences by physician specialty. Inpatient setting was associated with fewer recommended ASM prescriptions (vs. outpatient setting) (RR 0.82; 95% CI 0.75–0.89). There was no difference between emergency department and outpatient prescriptions.

**Conclusions:**

Understanding where/why less favorable ASM prescription may occur is important to target potential prescribing interventions. In this study, recommended ASM prescriptions were associated with patient, prescriber and setting characteristics. Notably, trainees prescribed recommended/neutral ASM more often, which underscores the importance of prescriber education in improving prescribing practices.

## 1. Introduction

Medical treatment is the primary modality of epilepsy treatment, and initiation is typically recommended at the time of diagnosis., [1] Established guidelines have existed for treatment of newly diagnosed epilepsy for nearly two decades., [2,3], Medical treatment is almost universally recommended at the time of epilepsy diagnosis[1] with current treatment recommendations centered on avoiding anti-seizure medication (ASM) adverse effects. Most ASMs appear to be similarly effective in treating seizures, with approximately two-thirds of newly diagnosed epilepsy being responsive to medication[4] and persons with epilepsy (PWE) commonly attributing declines in seizure frequency to their ASMs., [5] Despite these guidelines’ long existence, they are followed inconsistently., [6–8]

Initial choice of ASM is an important factor in determining patient-level outcomes in PWE., [9,10], Identification of populations that are at risk for suboptimal medication prescription is important to inform care delivery pathways and knowledge translation strategies. Accordingly, in this study, we sought to identify any patient, setting or practitioner characteristics associated with prescription of non-guideline adherent ASMs for persons with newly diagnosed epilepsy.

## 2. Methods

### 2.1. Study design and data source

We performed a retrospective observational cohort study of newly diagnosed epilepsy in the Mount Sinai Healthcare System (MSHS) during 2011–2019. The MSHS is a large, New York State-based multi-hospital healthcare system with hospitals throughout the New York City (NYC) metropolitan area. MSHS’s catchment area includes not only NYC but the surrounding areas of New Jersey, Connecticut and New York State. As such, MSHS’s patient population is remarkably diverse: geographically, linguistically, racially and socioeconomically. Structured data, obtained through the Mount Sinai Data Warehouse, were derived from the electronic medical records (EMR) of all MSHS. These data include demographics, orders, laboratory values, setting and provider information for every encounter within the MSHS.

### 2.2. Cohort identification

**Fig. 1.**
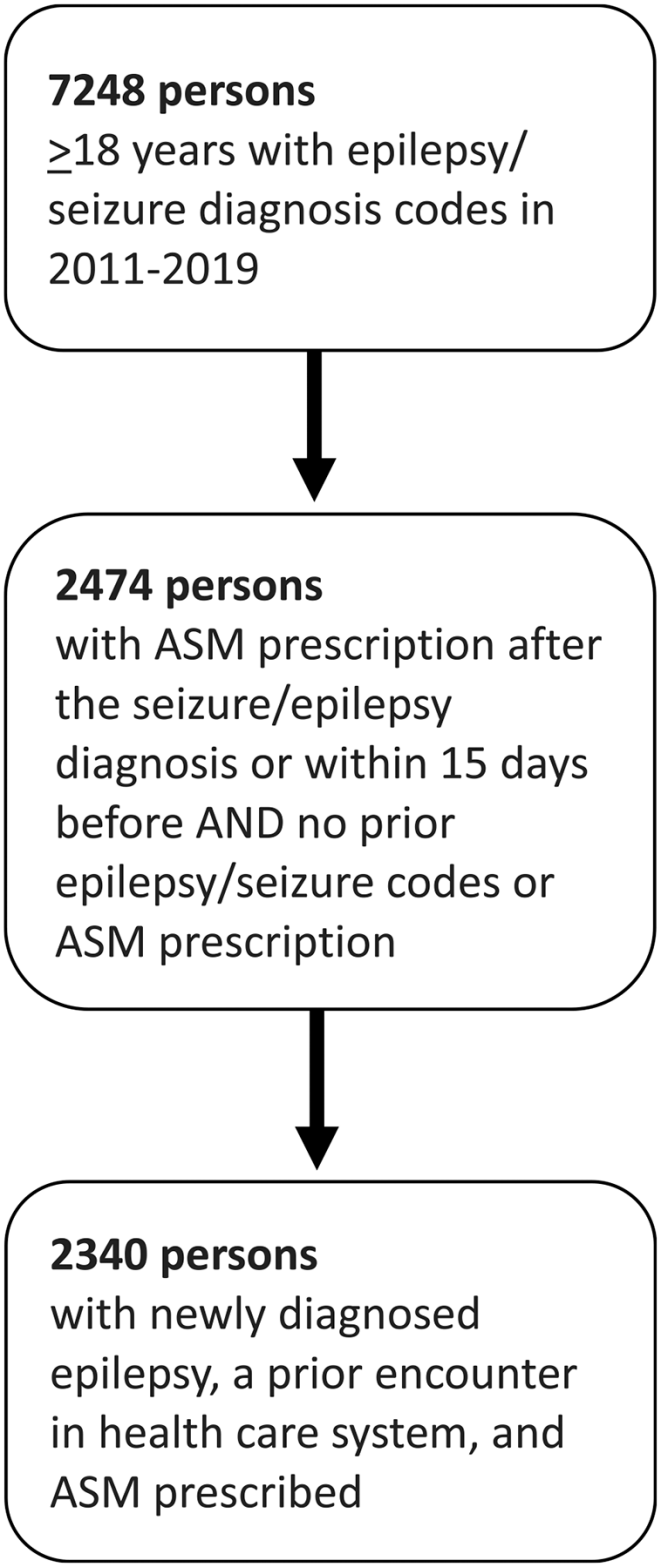
Cohort inclusion flow diagram.

Adults 18 years or older with newly diagnosed epilepsy at MSHS were identified for 2011–2019. We used a case-definition previously validated in US populations, including in our health system [11], for epilepsy: International Classification of Diseases, Ninth or Tenth Revision (ICD-9-CM or 10-CM) diagnosis codes for epilepsy or convulsion (345.xx/780.3x or G40.xx/R56.xx) and at least 30-day prescription for an ASM (lamotrigine, levetiracetam, zonisamide, carbamazepine, oxcarbazepine, esliscarbazepine, topiramate, lacosamide, brivaracetam, valproic acid, phenytoin, felbamate, phenobarbital, vigabatrin, rufinamide, clobazam, clonazepam, lorazepam, midazolam, diazepam, cannabidiol, gabapentin, pregabalin, acetazolamide, primidone, perampanel, ethosuximide, tiagabine hydrochloride, everolimus)., [12, 13], We required that the ASM was prescribed no sooner than 15 days before their first epilepsy diagnosis. Finally, to isolate instances of newly diagnosed epilepsy, consistent with the prior literature, we required that all participants had engagement with the healthcare system with at least one prior encounter in the MSHS system[14] and excluded anyone with any epilepsy-related diagnosis code or ASM prescription anytime for at least one year and up to 10 years before inclusion in the cohort (look--back period, see Fig. 1 for cohort flow chart and supplementary Figure 1 for cohort inclusion diagram).

### 2.3. Outcome measures

The primary outcome measured was first ASM prescribed. We initially categorized ASMs according to the American Academy of Neurology (AAN)/American Epilepsy Society (AES) guidelines for the “Treatment of New-onset Epilepsy.”[2] This categorization was supplemented with recommendations from international guidelines for the treatment of newly diagnosed epilepsy in adults., [15–17] We used: 1) “recommended” (lamotrigine, levetiracetam, zonisamide, gabapentin in adults >60), 2) “should be avoided” (phenytoin, felbamate, phenobarbital, vigabatrin, primidone, acetazolamide, perampanel, tiagabine hydrochloride, cannabidiol, everolimus, rufinamide, valproic acid in females age ≤45, or ethosuximide in adults aged ≥40), 3) “neutral” (topiramate, lacosamide, oxcarbazepine, carbamazepine, pregabalin, eslicarbazepine acetate, brivaracetam, ethosuximide in adults aged <40, gabapentin in adults aged <60, or valproic acid in males of any age and females aged >45), or 4) “benzodiazepines” (lorazepam, clonazepam, diazepam, midazolam, or clobazam) (Supplementary Table 1). In our analysis, the “recommended” and “neutral” categories were combined as these groups include medications that are widely believed to be effective, safe and well tolerated in most persons with newly diagnosed epilepsy. Similarly, “should be avoided” and benzodiazepines were combined as these medications are generally seen as inappropriate first-line chronic medications for epilepsy. When two or more ASMs were prescribed on the same date, individual charts were reviewed by an epileptologist (LJB) to determine which was intended to be used as a long-term medication for seizure prevention.

### 2.4. Covariates

Covariates at the patient, prescriber and setting level were selected a priori from clinical experience and literature review. Patient characteristics we examined included: age, sex, race, ethnicity, relationship status, preferred language, primary insurance at diagnosis, Charlson Comorbidity Index[18,19], as well as specific neurologic comorbidities often associated with seizures (stroke, Alzheimer’s disease and related dementia, brain tumor or traumatic brain injury). The neurologic comorbidities were defined using previously published ICD diagnostic codes (Supplementary Table 2)., [20] Charlson comorbidities were identified at or before the date of the initial epilepsy diagnosis code, [19, 21], and these comorbidities were transformed into a comorbidity index for each individual participant. Prescriber characteristics were extracted from the EMR and included provider type (advanced practice provider, physician trainee, physician or other/unspecified) and provider specialty (Emergency Medicine, Primary Care, Epilepsy, Neurology, Neurosurgery or other). Prescription setting was similarly extracted from the EMR encounter and was categorized by encounter location into outpatient, inpatient or emergency department (Table 1).

### 2.5. Statistical analysis

We described covariates of the study cohort stratified by grouped ASM categories (recommended/neutral vs. to be avoided/benzos), with categorical variables expressed as frequencies with proportions (percentage), and continuous variables as mean ± standard deviation. Chi-square tests, or when appropriate Fisher exact tests, were used to analyze categorical variables, and for post-hoc testing, the Holm-Bonferroni correction was applied to multiple comparisons. Continuous variables were assessed for normal distribution and were analyzed using T-tests. Due to the high prevalence of the outcome measure, we performed Poisson multiple regression using robust standard errors to identify the relationship between first ASM choices and patient, prescriber and setting characteristics and to report relative risk ratios (RR) and 95 % confidence intervals (CIs) for these analyses. Our models included the covariates described above. A two-sided p-value < 0.05 was considered significant. To ensure the robustness of our findings we performed a sensitivity analysis that removed the ‘neutral’ ASM category, comparing ‘recommended’ vs. ‘to be avoided/benzodiazepines’. All analyses were performed using Statistical Analysis Software version 9.4 (SAS Institute Inc, Cary, NC, USA).

### 2.6. Data availability statement

Supporting data include confidential medical information. These data are available upon reasonable request to qualified researchers for non-commercial research purposes subject to a data use agreement.

### 2.7. Standard protocol approvals, registrations and patient consent

The Icahn School of Medicine at Mount Sinai Institutional Review Board has approved this project and waived need for individual participant consent.

## 3. Results

We identified 2340 people with new-onset epilepsy in 2011–2019 who were prescribed an ASM within one year (Fig. 1). Participants were engaged within the MHSH healthcare system with 72 % having had a visit in the year prior to their epilepsy diagnosis and 79 % having had a visit at least two years prior to their epilepsy diagnosis. The cohort was 48.3 % female and 54.6 years old on average. Neurologic comorbidities associated with epilepsy were uncommon: Alzheimer’s and related dementias (not reportable due to small cell size (N.R.)) brain tumor (1.1 %), stroke (4.4 %) and traumatic brain injury (N.R.) (Table 1). The most prescribed ASM was levetiracetam (*n* = 1065, 45.5 %). Overall, more than half of the cohort (60.7 %) were prescribed a neutral or recommended ASM, and these prescriptions most occurred in the outpatient setting (50.3 %) followed by in-patient (32.9 %) and emergency department (16.8 %). Prescribers were most frequently physicians (67.0 %) or trainee physicians (20.4 %) and less frequently advanced practice providers (11.3 %). Neurology (excluding epilepsy) was identified as prescribing specialty in 15 % and emergency medicine in 11.6 % of patients. The majority of prescriptions (53.8 %) were not written by neurology, nor neurosurgery, nor internal medicine/pediatrics/family practice. The potentially inappropriate ASM medication prescriptions were most often phenytoin or a benzodiazepine. This was true across several studied characteristics including different prescribers, settings and patient ages (see Table 2). Of note, only 12 females between the ages of 18–45 were prescribed valproic acid.

**Fig. 2.**
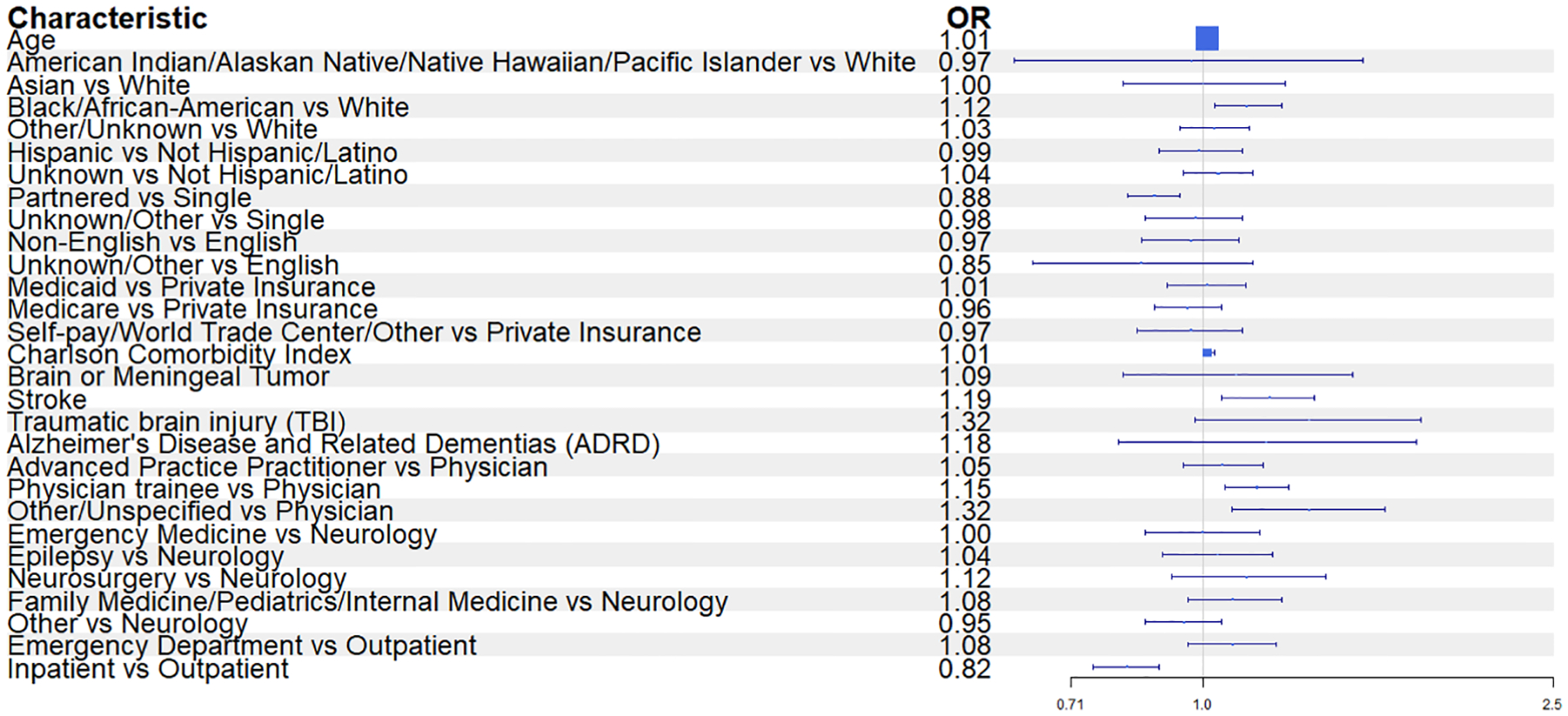
Forrest Plot of Multivariable Poisson regression for risk ratios modelling the probability of being prescribed a neutral/recommended ASM (*N* = 2340).

Multivariable Poisson models were built to examine the association between first drug prescribed and patient, physician and setting characteristics (Fig. 2, Supplementary Table 3). These models showed that prescription of recommend or neutral categories of ASM as the first medication for epilepsy was associated with a number of patient characteristics including increasing age (relative risk (RR) 1.01; 95 % confidence interval (CI) 1.00–1.01), Black race (RR 1.12; 95 % CI 1.03–1.23), being in a relationship (vs. divorced/separated/single or widowed) (RR 1.13; 95 % CI 1.06–1.22)) and a history of stroke (RR 1.19; 95 % CI 1.05–1.34). Prescriptions from physician trainees (vs. non-trainees) were more likely to align with recommended/neutral ASMs (RR 1.15; 95 % CI 1.06–1.25), but no differences in prescribing were seen by prescriber specialty. Inpatient setting was associated with fewer recommended ASM prescriptions (vs. outpatient setting RR 0.82; 0.75–0.89). There was no difference between emergency department and outpatient prescriptions. To ensure the robustness of our findings we performed a sensitivity analysis that removed the ‘neutral’ category and compared ‘recommended’ to ‘should be avoided/benzodiazepines’, which showed similar results (see Supplementary Table 4).

## 4. Discussion

In this study, we show that preferred ASM prescription for newly diagnosed epilepsy is associated with specific patient, prescriber and setting characteristics. These findings suggest that potential interventions to improve prescribing can be targeted to groups at higher risk of receiving or prescribing suboptimal ASMs. Reassuringly, this work continues to show that most adults are started on recommended first-line medications, with levetiracetam prescribed to nearly half (45.5 %) of the adults in this cohort. There remains, however, a considerable proportion of patients started on suboptimal ASMs, which may be associated with a number of adverse health outcomes., [2,9,10,22–25],

Our final model showed an increased likelihood of recommend or neutral prescription in groups conceivably at higher risk of inappropriate prescription, including increased age., [26] This may reflect more careful attention to possible side effects of ASM in older adults encouraged by prescribing/deprescribing initiatives like the American Geriatrics Society’s Beers Criteria^®^., [27] Our study identified no differences in first ASM prescribed by ethnicity or insurance type, but being in a relationship increased the likelihood of being prescribed a recommended or neutral drug. This may suggest the role of even informal care partners in improving health outcomes, which has previously been seen in other neurologic disorders including dementia., [28,29], Black race was also interestingly associated with higher likelihood of appropriate prescription which is different than what has been found in prior studies., [30] This may be in part due to residual confounding as this patient population may be more likely to be seen by trainees and/or in the ED setting where the likelihood of recommended prescribing is higher. The other patient characteristic associated with increased likelihood of recommended or neutral ASM prescription was persons with stroke, which again may reflect that stroke care is generally protocolized and evidence-based[31] with prescribers treating these patients perhaps being more likely to be up to date on current treatment guidelines.

Interestingly, our examination of prescriber type showed that trainee physicians were more likely to be the prescriber of recommended or neutral ASMs as compared to physicians who have completed training. Similarly, prescribers that were uncategorized in our system were more likely to prescribe recommended or neutral medications. This improved guideline-adherence is different from prior research that had showed trainee prescribers having higher prescribing errors (e.g. missed drug-drug interactions)., [32] Trainee physicians should be closer to having received education about current prescribing guidelines, and this finding of improved first prescription in the trainee group may highlight the importance of continuing medical education (CME) for prescribers in improving prescribing practices.

Unsurprisingly, differences exist in ASM prescription by setting, with outpatient and emergency department prescribing being associated with increased likelihood of recommend or neutral ASM as compared to inpatient. This finding likely reflects the severity of presentation that may require alternative ASM medication, especially one that can be delivered intravenously. It does, however, highlight a potential opportunity for thoughtful deprescribing around the time of discharge. Better understanding of deprescribing of ED/inpatient ASMs would an important future direction focused on the inpatient setting.

Finally, the most frequently prescribed potentially inappropriate ASMs were either benzodiazepines or phenytoin. These are both medications with long document adverse effects[2,9,10,22–25], which may suggest that current educational and dissemination practices are insufficient.

### 4.1. Limitations

This study is unique as it uses a large multi-hospital health system with a diverse, multi-state catchment to examine the association between ASM prescription and patient, prescriber and setting characteristics. There are, however, several possible limitations. First, our study assessed the population during 2011–2019, and may not fully reflect the current experience. Second, the structured EMR data does not allow for a detailed examination of socioeconomic status, attitudes towards healthcare, adherence to medication, functional status, or epilepsy severity—all of which might impact decisions as to which medications to prescribe. Some comorbidities that might influence prescribing, such as depression and migraine were not included in our analysis as these conditions are not well identified using ICD coding. In addition, while our health system’s EMR is linked to most institutions in our region, and although we included only persons with prior encounters in our health care system to help reduce any misclassification of prevalent epilepsy, we are likely to have misclassified some newly diagnosed epilepsy cases. This EMR-based dataset, however, provides provider and setting level-information as well as data on inpatient prescriptions not available in US-administrative data generated for insurance billing purposes.

## 5. Conclusions

We present data on patient, prescriber and setting characteristics associated with ASM prescription for newly diagnosed epilepsy. These findings highlight our ability to target potential prescribing interventions to patients, prescribers and settings most likely to be associated with less favorable ASM prescription. Among other key findings, trainees prescribed recommended/neutral ASM more often, which may indicate the importance of prescriber education in improving prescribing practices.

## Supplementary Material

Supplementary material

Supplementary material associated with this article can be found, in the online version, at doi:10.1016/j.seizure.2026.02.007.

## Figures and Tables

**Table 1:** Baseline characteristics of persons with newly diagnosed epilepsy in by category of first ASM prescribed.

Characteristic	Total (*N* = 2340)	Neutral/Recommended (*N* = 1420)	Benzos/To be Avoided (*N* = 920)	P-value
Age at diagnosis (yr.)	54.6 ± 20.1	56.6 2 ± 20.5	51.5 1 ± 19.2	**<0.0001**
Sex				**0.01**
Male	1209 (51.7)	701 (49.4)	508 (55.2)	
Female	1131 (48.3)	719 (50.6)	412 (44.8)	
Race				0.6
American Indian/Alaskan Native/Native Hawaiian/Pacific Islander	819 (35)	493 (34.7)	326 (35.4)	
Asian	594 (25.4)	377 (26.5)	217 (23.6)	
Black/African-American	54 (2.3)	33 (2.3)	21 (2.3)	
Other/Unknown	N.R.	N.R.	N.R.	
White	858 (36.7)	509 (35.8)	349 (37.9)	
Ethnicity				0.5
Hispanic	428 (18.3)	249 (17.5)	179 (19.5)	
Not Hispanic or Latino	1402 (59.9)	855 (60.2)	547 (59.5)	
Unknown	510 (21.8)	316 (22.3)	194 (21.1)	
Relationship Status				**<0.0001**
Partnered	711 (30.4)	477 (33.6)	234 (25.4)	
Single	1459 (62.4)	834 (58.7)	625 (67.9)	
Unknown/Other	170 (7.3)	109 (7.7)	61 (6.6)	
Preferred Language				0.3
English	2120 (90.6)	1291 (90.9)	829 (90.1)	
Non-English	176 (7.5)	107 (7.5)	69 (7.5)	
Unknown/Other	44 (1.9)	22 (1.5)	22 (2.4)	
Insurance at diagnosis				0.1
Self-Pay/WTC/Other	198 (8.5)	114 (8)	84 (9.1)	
Medicaid	473 (20.2)	273 (19.2)	200 (21.7)	
Medicare	1082 (46.2)	683 (48.1)	399 (43.4)	
Private Insurance	587 (25.1)	350 (24.6)	237 (25.8)	
Charlson Comorbidity Index	0.8 1 ± 1.7	0.9 ± 1.7	0.7 ± 1.6	0.05
Brain/meningeal tumor	23 (1.1)	N.R.	N.R.	0.7
Stroke	103 (4.4)	77 (5.4)	26 (2.8)	**0.003**
Traumatic brain injury	N.R.	N.R.	N.R.	0.2
Alzheimer and other dementias	N.R.	N.R.	N.R.	0.5
Health care provider				0.5
Advanced practice provider	265 (11.3)	162 (11.4)	103 (11.2)	
Physician trainee	477 (20.4)	308 (21.7)	169 (18.4)	
Physician	1567 (67.0)	926 (65.2)	641 (69.7)	
Other/Unspecified	31 (1.3)	24 (1.7)	N.R.	
Provider Specialty				0.3
Emergency Medicine	272 (11.6)	165 (11.6)	107 (11.6)	
Family Medicine/Pediatrics/Internal Medicine	223 (9.5)	147 (10.4)	76 (8.3)	
Epilepsy	175 (7.5)	106 (7.5)	69 (7.5)	
Neurology	352 (15)	217 (15.3)	135 (14.7)	
Neurosurgery	58 (2.5)	40 (2.8)	N.R.	
Other	1260 (53.8)	745 (52.5)	515 (56)	
Setting				**0.01**
Outpatient	1177 (50.3)	735 (51.8)	442 (48)	
Inpatient	770 (32.9)	433 (30.5)	337 (36.6)	
Emergency Department	393 (16.8)	252 (17.7)	141 (15.3)	

Note: Continuous variables are expressed as mean ± standard deviation and analyzed using T-tests; categorical variables are expressed as frequency (percent) and are analyzed using Chi-square test or when appropriate Fisher exact test; *Significant by post-hoc testing using Holm-Bonferroni correction. N. R.= non-reportable for cell size less than 20. Relationship status: Partnered included those who reported being married, in a civil union, having a significant other or life partner. Single refers to those who reported being divorced, separated, single or widowed.

**Table 2:** Top three most prescribed potentially inappropriate first ASM by setting, prescriber or patient characteristic.

Prescription Setting	
Emergency Department	Phenytoin (8.5 %), lorazepam (7.1 %), diazepam (3.1 %)
Outpatient	Phenytoin (4.7 %), clonazepam (4.1 %), lorazepam (3.1 %)
Inpatient	Lorazepam (20.4 %), phenytoin (6.3 %), midazolam (4.5 %)
Patient Age	
18–59 years	Lorazepam (8.6 %), phenytoin (5.3 %), clonazepam (4.1 %)
60+ years	Lorazepam (10.6 %), phenytoin (6.7 %), clonazepam (2.1 %)
Physician trainee status	
Trainee physician	Lorazepam (11.5 %), phenytoin (5.2 %), clonazepam (2.1 %)
Non-trainee physician	Lorazepam (9.2 %), phenytoin (5.8 %), clonazepam (3.7 %)
