# Supplementary material for: Characteristics associated with first anti-seizure medication prescribed in a cohort of adults with newly diagnosed epilepsy
Source: Seizure. Author manuscript; Available in PMC 2026 Apr 17. (PMC13089088; doi:10.1016/j.seizure.2026.02.007)

**Supplementary Table 1: Categorization of Anti-seizure Medications**

| Recommended | lamotrigine, levetiracetam, zonisamide, gabapentin in adults >60 |
| --- | --- |
| Should be avoided | phenytoin, felbamate, phenobarbital, vigabatrin, primidone, acetazolamide, perampanel, tiagabine hydrochloride, cannabidiol, everolimus, rufinamide, valproic acid in females age <=45, or ethosuximide in adults aged >=40 |
| Neutral | topiramate, lacosamide, oxcarbazepine, carbamazepine, pregabalin, eslicarbazepine acetate, brivaracetam, ethosuximide in adults aged <40, gabapentin in adults aged <60, or valproic acid in males of any age and females aged >45 |
| Benzodiazepines | lorazepam, clonazepam, diazepam, midazolam, or clobazam |

**Supplementary Table 2: ICD-CM codes used for identification of comorbid disorders.**

|  |  | **ICD9** | **ICD10** |
| --- | --- | --- | --- |
| **Neurologic Comorbidities** | | | |
| **Brain/meningeal tumor** |  | 191.x, 192.1, 198.3, 198.4 | C71.9, C79.31, C79.32, C70 |
| **Stroke** |  | 430-438 | I60-I63, I67.82, I69 |
| **Traumatic brain injury** |  | 310.2, 800-801, 802.6, 802.7, 803.x-804.x, 850.0-854.1, 959.01, 907.0, 950.1, 950.3 | S02.0, S02.1, S02.3, S02.4, S02.8, S02.9, S04, S06, S07, S08, S09.0x, S09.2x, S09.3x, S09.9x |
| **Alzheimer and other Dementias** |  | 46.x, 290.x, 294.x, 331.x | F02, A81.0x, G31.0x, G31.1, G31.2, G31.83, G31.85, G31.89, G31.9, F03, F01, F04, G30 |

ICD-International Classification of Diseases

**Supplementary Table 3. Multivariable Poisson regression for risk ratios modelling the probability of being prescribed a neutral/recommended ASM (N = 2340).**

|  |  | **RR** | **95% CI** | | **P-Value** |
| --- | --- | --- | --- | --- | --- |
| Age at diagnosis (increasing years) | | 1.01 | 1.00 | 1.01 | **<0.0001** |
| Race | | | | | |
|  | White | Reference | | | |
|  | American Indian/Alaskan Native/Native Hawaiian/Pacific Islander | 0.97 | 0.61 | 1.52 | 0.9 |
|  | Asian vs White | 1.00 | 0.81 | 1.24 | 1 |
|  | Black/African-American | 1.12 | 1.03 | 1.23 | **0.01** |
|  | Other/Unknown | 1.03 | 0.94 | 1.13 | 0.5 |
| Ethnicity | | | | | |
|  | Not Hispanic/Latino | Reference | | | |
|  | Hispanic | 0.99 | 0.89 | 1.11 | 0.9 |
|  | Unknown | 1.04 | 0.95 | 1.14 | 0.4 |
| Relationship Status | |  |  |  |  |
|  | Partnered | Reference | | | |
|  | Single | 0.88 | 0.82 | 0.94 | **0.0002** |
|  | Unknown/Other | 0.98 | 0.86 | 1.11 | 0.7 |
| Language Spoken | |  |  |  |  |
|  | English | Reference | | | |
|  | Non-English | 0.97 | 0.85 | 1.10 | 0.6 |
|  | Unknown/Other | 0.85 | 0.64 | 1.14 | 0.3 |
| Insurance Type | |  |  |  |  |
|  | Private Insurance | Reference | | | |
|  | Medicaid | 1.01 | 0.91 | 1.12 | 0.9 |
|  | Medicare | 0.96 | 0.88 | 1.05 | 0.4 |
|  | Self-pay/World Trade Center/Other | 0.97 | 0.84 | 1.11 | 0.7 |
| Charlson Comorbidity Index | | 1.01 | 1.00 | 1.03 | 0.1 |
| Neurologic comorbidity | |  |  |  |  |
|  | Brain or Meningeal Tumor | 1.09 | 0.81 | 1.48 | 0.6 |
|  | Stroke | 1.19 | 1.05 | 1.34 | **0.01** |
|  | Traumatic brain injury (TBI) | 1.32 | 0.98 | 1.77 | 0.06 |
|  | Alzheimer's Disease and Related Dementias (ADRD) | 1.18 | 0.80 | 1.75 | 0.4 |
| Health care provider | |  |  |  |  |
|  | Physician | Reference | | | |
|  | Advanced Practice Practitioner (APP) | 1.05 | 0.95 | 1.17 | 0.3 |
|  | Physician trainee | 1.15 | 1.06 | 1.25 | **0.001** |
|  | Other/Unspecified | 1.32 | 1.08 | 1.61 | **0.01** |
| Specialty | |  |  |  |  |
|  | Neurology | Reference | | | |
|  | Emergency Medicine | 1.00 | 0.86 | 1.16 | 1 |
|  | Epilepsy | 1.04 | 0.90 | 1.20 | 0.6 |
|  | Neurosurgery | 1.12 | 0.92 | 1.38 | 0.3 |
|  | Family Medicine/Pediatrics/Internal Medicine | 1.08 | 0.96 | 1.23 | 0.2 |
|  | Other | 0.95 | 0.86 | 1.05 | 0.3 |
| Setting | |  |  |  |  |
|  | Outpatient | Reference | | | |
|  | Emergency Department | 1.08 | 0.96 | 1.21 | 0.2 |
|  | Inpatient | 0.82 | 0.75 | 0.89 | **<0.0001** |

Note: RR, risk ratio; CI, confidence interval. Relationship status: Partnered included those who reported being married, in a civil union, having a significant other or life partner. Single refers to those who reported being divorced, separated, single or widowed.

**Supplementary Table 4: Multivariable Poisson regression for risk ratios modelling the probability of being prescribed a recommended ASM (N=2228)**

|  |  | **RR** | **95% CI** | | **P-Value** |
| --- | --- | --- | --- | --- | --- |
| Age at diagnosis (increasing years) |  | 1.01 | 1.00 | 1.01 | **<0.0001** |
| Race |  |  |  |  |  |
|  | White | Reference |  |  |  |
|  | American Indian/Alaskan Native/Native Hawaiian/Pacific Islander | 0.77 | 0.42 | 1.41 | 0.4 |
|  | Asian | 0.97 | 0.78 | 1.22 | 0.8 |
|  | Black/African-American | 1.13 | 1.03 | 1.24 | **0.01** |
|  | Other/Unknown | 1.01 | 0.91 | 1.12 | 0.8 |
| Ethnicity |  |  |  |  |  |
|  | Not Hispanic/Latino | Reference |  |  |  |
|  | Hispanic | 1.00 | 0.88 | 1.12 | 0.9 |
|  | Unknown | 1.05 | 0.96 | 1.16 | 0.3 |
| Relationship Status |  |  |  |  |  |
|  | Partnered | Reference |  |  |  |
|  | Single | 0.86 | 0.80 | 0.93 | **0.0001** |
|  | Unknown/Other | 0.98 | 0.85 | 1.12 | 0.7 |
| Language Spoken |  |  |  |  |  |
|  | English | Reference |  |  |  |
|  | Non-English | 0.97 | 0.85 | 1.11 | 0.7 |
|  | Unknown/Other | 0.87 | 0.65 | 1.18 | 0.4 |
| Insurance Type |  |  |  |  |  |
|  | Private Insurance | Reference |  |  |  |
|  | Medicaid | 0.99 | 0.88 | 1.1 | 0.8 |
|  | Medicare | 0.94 | 0.86 | 1.0 | 0.2 |
|  | Self-pay/World Trade Center/Other | 0.94 | 1.09 | 0.81 | 0.4 |
| Charlson Comorbidity Index |  | 1.01 | 0.99 | 1.0 | 0.2 |
| Neurologic comorbidity |  |  |  |  |  |
|  | Brain or Meningeal Tumor |  |  |  |  |
|  | Stroke | 1.21 | 1.06 | 1.37 | **0.004** |
|  | Traumatic brain injury (TBI) | 1.30 | 0.95 | 1.78 | 0.1 |
|  | Alzheimer's Disease and Related Dementias (ADRD) | 1.22 | 0.82 | 1.82 | 0.3 |
| Health care provider |  |  |  |  |  |
|  | Physician | Reference |  |  |  |
|  | Advanced Practice Practitioner (APP) | 1.08 | 0.97 | 1.21 | 0.2 |
|  | Physician trainee | 1.18 | 1.08 | 1.3 | **0.0004** |
|  | Other/Unspecified | 1.30 | 1.04 | 1.6 | **0.02** |
| Specialty |  |  |  |  |  |
|  | Neurology | Reference |  |  |  |
|  | Emergency Medicine | 1.03 | 0.88 | 1.20 | 0.7 |
|  | Epilepsy | 1.06 | 0.91 | 1.24 | 0.4 |
|  | Neurosurgery | 1.16 | 0.94 | 1.42 | 0.2 |
|  | Family Medicine/Pediatrics/Internal Medicine | 0.91 | 0.80 | 1.04 | 0.2 |
|  | Other | 1.05 | 0.95 | 1.17 | 0.3 |
| Setting |  |  |  |  |  |
|  | Outpatient | Reference |  |  |  |
|  | Emergency Department | 1.08 | 0.96 | 1.22 | 0.2 |
|  | Inpatient | 0.80 | 0.73 | 0.9 | **<0.0001** |

**Supplementary Figure 1.**


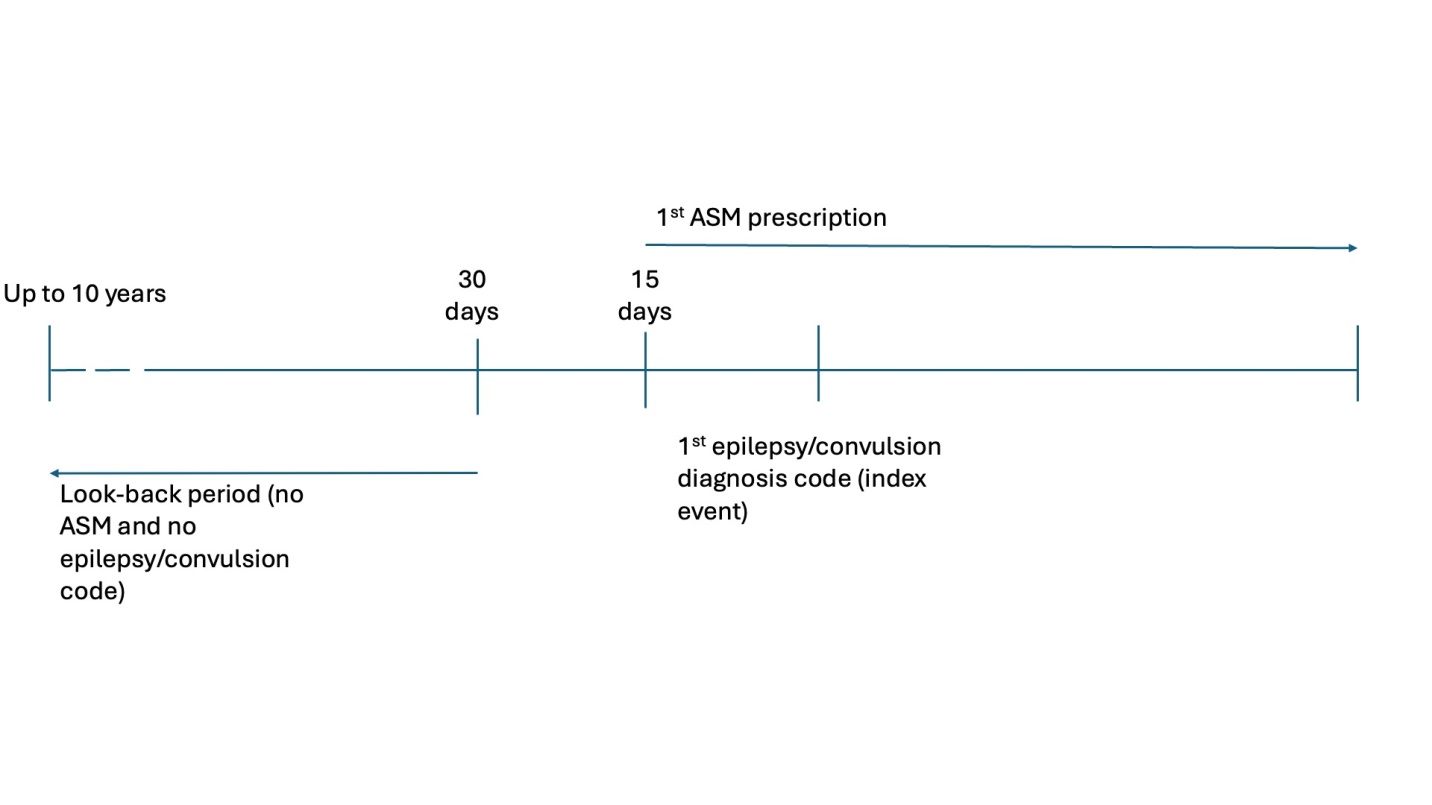

Supplement: Supplementary material [file NIHMS2163155-supplement-Supplementary_material.docx]
